# Identification of Fatty Acid Desaturases in Maize and Their Differential Responses to Low and High Temperature

**DOI:** 10.3390/genes10060445

**Published:** 2019-06-12

**Authors:** Xunchao Zhao, Jinpeng Wei, Lin He, Yifei Zhang, Ying Zhao, Xiaoxuan Xu, Yulei Wei, Shengnan Ge, Dong Ding, Meng Liu, Shuren Gao, Jingyu Xu

**Affiliations:** Key Lab of Modern Agricultural Cultivation and Crop Germplasm Improvement of Heilongjiang Province, College of Agriculture, Heilongjiang Bayi Agricultural University, Daqing 163319, China; zhaoxunchao2017@163.com (X.Z.); weijp81@163.com (J.W.); linlinhe65@sina.com (L.H.); byndzyf@163.com (Y.Z.); zhaoying0209@hotmail.com (Y.Z.); xuxiaoxuan3344@163.com (X.X.); wyl8390@163.com (Y.W.); geshnan@163.com (S.G.); nxdingdong@yahoo.com (D.D.); nxliumeng@yahoo.com (M.L.)

**Keywords:** Maize (*Zea mays* L.), fatty acid desaturases (FADs), bioinformatics, expression analysis, cold stress, heat stress

## Abstract

Plant fatty acid desaturases (FADs) catalyze the desaturation of fatty acids in various forms and play important roles in regulating fatty acid composition and maintaining membrane fluidity under temperature stress. A total of 30 FADs were identified from a maize genome, including 13 soluble and 17 membrane-bound FADs, which were further classified into two and five sub-groups, respectively, via phylogenetic analysis. Although there is no evolutionary relationship between the soluble and the membrane-bound FADs, they all harbor a highly conserved FA_desaturase domain, and the types and the distributions of conserved motifs are similar within each sub-group. The transcriptome analysis revealed that genes encoding FADs exhibited different expression profiles under cold and heat stresses. The expression of *ZmFAD2.1*&*2.2*, *ZmFAD7,* and *ZmSLD1*&*3* were significantly up-regulated under cold stress; moreover, the expression of *ZmFAD2.1*&*2.3* and *ZmSLD1*&*3* were obviously down-regulated under heat stress. The co-expression analysis demonstrated close correlation among the transcription factors and the significant responsive FAD genes under cold or heat stress. This study helps to understand the roles of plant FADs in temperature stress responses.

## 1. Introduction

Plant lipids constitute the lipid double layer structure of biofilms and thus are the main components of various cell membranes [1]. Fatty acids are essential constituents in the architecture of biomembranes, and the species and the unsaturation of fatty acid chains are important for membrane functions in plants [2,3,4]. The lipid unsaturation affects the membrane fluidity and therefore is vital for plants to cope with temperature stresses [5]. The catalysts that can form unsaturated fatty acids by inserting double bonds in fatty acid are called fatty acid desaturases (FADs) [6]. Thus far, two types of FADs have been identified from animals, plants, fungi, and algae; one is soluble stearoyl-ACP (Acyl-carrier protein) desaturase (FAB2, also known as SAD), and another is membrane-bound FAD, which desaturates fatty acids that are esterified to various kind of lipids [7,8,9,10].

De novo synthesis of plants fatty acids occurs mainly in plastids, and the process uses acetyl-CoA as a substrate and relies on the participation of ACP [11]. Catalyzed by the fatty acid synthase (FAS) complex, malonyl-CoA binds to ACP, and the acyl chain undergoes a continuous condensation reaction in the form of two carbon atoms per cycle to produce 16:0-ACP and 18:0-ACP [12]. The soluble stearoyl-ACP desaturase (FAB2) catalyzes the desaturation of 18:0-ACP at the Δ9 position to form 18:1-ACP, which is the key step of regulating the level of unsaturated fatty acid (FA) in cells [13]. The resulting 18:1-ACP can either enter the prokaryotic glycerolipid pathway or be hydrolyzed to free fatty acid and exported to cytosol and activated to CoA esters for triacylglycerol (TAG) and phospholipids synthesis in the endoplasmic reticulum (ER) [14].

In plants, there are two spatially independent pathways for the synthesis of glycerolipids, which are found in chloroplasts and the ER, respectively, and are often referred to as prokaryotic and eukaryotic pathways [1]. In the ER/eukaryotic pathway, acyl groups esterified to phosphatidylcholine (PC) are the site for membrane-bound FADs [14]. FAD2 and FAD3 convert PC-bound oleate (18:1) to linoleate (18:2) and then linolenate (18:3), respectively [15,16]. The remaining FAD enzymes (FAD4–8) are all chloroplast-localized and catalyze desaturation reactions in the prokaryotic pathway with unique substrate specificity [17,18]. For catalysis, FAD5–8 uses FAs that are esterified to plastidial lipids, such as phosphatidyl glycerol (PG), sulfoquinovosyl diacylglycerol (SQDG), and digalactosyldiacylglycerol (DGDG), resulting in high linolenate (18:3) lipid species [19]. FAD4 is unique because it introduces a *trans* double bond into PG (16:0, 18:1) that leads to the generation of PG (t16:1, 18:1) [20].

Studies on *FAD* genes in a range of plant species have revealed their active responses to cold or heat stresses. An increased production of unsaturated fatty acids, such as 18:3, is associated with cold stress responses in plants [4]. In *Pinus radiate*, accumulation of saturated fatty acids to reduce membrane fluidity was found under high temperature stress [21]. Mutation in *FAD2* and *FAD6* in *Arabidopsis* reduced the level of polyunsaturated fatty acids and resulted in sensitivity to low temperature [22,23]. In oil palm, the promoter of *FAD8* was evidently induced under cold [24]. Over-expressing *FAD8* alone was enough to enhance heat sensitivity. Meanwhile, over-expressing *FAD3* and *FAD8* improved drought tolerance in tobacco [25]. In rice, the *FAD8* knock-out further decreased membrane fluidity under low temperature [26].

Sphingolipids are also important components of cell membranes and are only detected in eukaryotic cells [27]. Sphingolipids are produced through the condensation of serine and palmitoyl-CoA in the ER catalyzed by serine-palmitoyl transferase (SPT) [27]. The resulting long-chain bases (LCBs) can undergo several modifications in plants, such as Δ4-hydroxylation, Δ4-desaturation, and Δ8-desaturation [18]. The membrane-bound sphingolipid Δ4-desaturases (DES) uses sphingolipid-bound FAD to catalyze the formation of d18:1∆4 sphingolipid; however, sphingoid LCB Δ8 desaturase (SLD) conducts the d18:1Δ8 sphingolipid formation [18,20]. Researchers have shown that Δ8 desaturation of LCBs in both *cis/trans*-configurations in plants might affect the cold resistance of plants [18,28]. In cotton (*Gossypium raimondii*), *GrSLD2/4/5* and *GrDSD1* genes were found obviously up-regulated under cold conditions [29].

Although the identification of FADs has been reported in a number of plant species, the comprehensive characterization and analysis of the complicated FAD families are still needed [30,31]. In this study, genetic information, evolutionary relationship, gene structure, conserved domain analysis, chromosome localization, and stress responses of the FADs were investigated in maize. Our data provide helpful information on understanding the roles of plants FADs in temperature stress responses.

## 2. Materials and Methods

### 2.1. Identification of the FAD and FAB2 Gene Families in Maize

*Arabidopsis FAD* and *FAB2* genes sequence data were obtained from Tair database (http://www.arabidopsis.org/). To identify all candidate *FAD* and *FAB2* genes in maize, systematic BLASTp (https://blast.ncbi.nlm.nih.gov/Blast.cgi) searches were performed against the maize reference genome (https://maizegdb.org/) and the NCBI database using the published sequences of *Arabidopsis* and other plants *FAD* and *FAB2* genes as queries. The screening criteria was E value (<10^−10^) and protein length > 200 aa. The candidate *ZmFAD* and *ZmFAB2* genes were confirmed at the online Pfam (http://pfam.xfam.org/) [32] and SMART (http://smart.embl-heidelberg.de) [33] website using the specific gene name and the typical functional domains of FADs and FAB2. A total of 30 non-redundant candidate *ZmFAD* and *ZmFAB2* genes were retained for further analysis. The conserved domains, including FA_desaturase, DES-lipids, cyt-b5, and TMEM189_B_Dmain domain, were predicted using the Pfam and the SMART databases, and their distribution was visualized by IBS software version 2.0 [34].

### 2.2. Sequence Alignment and Phylogenetic Tree Construction

The full-length amino acid sequences of FADs from maize (*Zea mays*), *Arabidopsis* (*Arabidopsis thaliana*), rice (*Oryza sativa*), sorghum (*Sorghum bicolor*), and soybean (*Glycine max*) were obtained from an online database (https://www.ncbi.nlm.nih.gov/) using NCBI BLASTp tools. The multiple sequence alignments of amino acid sequences of these FADs and FAB2s were conducted by the online ClustalX software [35], and the phylogenetic trees were constructed separately for the membrane-bound FAD and the soluble FAB2 using the Neighbor-Joining method with the bootstrap values set at 1000 replicates.

### 2.3. Characteristics Gene Structure and Synteny Analysis

The accession numbers, the coding sequence length, the amino acid numbers, and the chromosomal localization of maize FADs were obtained from the Maize-Sequence database. The physical and the chemical properties, including molecular formula, molecular weight, and isoelectric point, were obtained from the Expasy website (http://web.expasy.org/cgi-bin/protparam/protparam) [36]. The exon/intron structures of the maize *FAD* and *FAB2* genes were unveiled at the Gene Structure Display Server (GSDS) (http://gsds.cbi.pku.edu.cn/index.php) [37]. The syntenic blocks among maize and rice genes were generated according to the Plant Genome Duplication Database.

### 2.4. Expression Profiles of Maize Fatty Acid Desaturase Genes in Diverse Tissues

The expression data of maize *FAD* and *FAB2* genes at different tissues and developmental stages were acquired from the online maize eFP database [38]. The heat maps representing the gene expression intensities were generated, and cluster analysis was completed by Tree View version 1.6 software.

### 2.5. Expression Profiles of the Maize FAD and FAB2 Genes in Response to Temperature Stresses

The gene expression data of maize *FAD* and *FAB2* genes under cold or heat stress treatments were obtained from the NCBI sequence-reading archive (SRA). The maize transcriptome data of leaves from 2-week old maize seedlings (variety He-344) under cold (5 °C, 3 days) treatment were originally generated by our lab and uploaded to NCBI with an accession number of SRX2672484 [39]. The transcriptome data of the aerial parts of 2-week old maize seedlings (variety B73) under heat stress (50 °C, 4 h) were reported by Makarevitch et al. (2015), and the accession numbers are SRR1238715, SRR1819196, and SRR1819198 [40]. The expression profiles of the differentially expressed genes were presented, and a Venn diagram showing overlapping genes under cold/heat stresses was depicted.

### 2.6. Co-Expression of FADs and Transcription Factors under Temperature Stresses

The transcription factors (TFs) that responded to cold or heat stress were screened from the cold or heat transcriptome databases. The strong cold/heat responsive genes were named as the “guide genes” and were used as the inquiry to explore the co-expression relationships with the corresponding transcription factors. The correlation coefficients were computed based on the expression data of the “guide genes” and the transcription factors (from the cold or the heat transcriptome databases). The co-expression relationship was constructed when the Pearson correlation was higher than 0.97, and the co-expression networks were built for those obviously co-expressed genes using the Perl script (default value 0.6). Co-expression visualization was achieved using the program Cytoscape v 3.4.10 [41].

### 2.7. Quantitative Real-Time RT-PCR Analysis

In order to further evaluate the reliability of transcriptome data under cold and heat stress, qRT-PCR analysis was conducted. Cold and heat treatments were conducted as described in the transcriptome studies [39,40]. The maize seedlings were grown in a mixture of vermiculite and soil (1:1, *v*/*v*), and the growth chamber was set at 24 °C and 16 h/8 h (light/dark) daily photoperiodic cycle. Two-week old maize seedlings were subjected to cold (5 °C for 3 days, maize variety He-344) and heat (50 °C for 4 h, maize variety B73) treatments, respectively. The leaf samples were collected after treatment, total RNA was extracted using Trizol reagent, and cDNA was synthesized using the ReverTra Ace qPCR RT Master Mix (TOYOBO, Osaka, Japan). qRT-PCR was performed in a 96-well plate using a SYBR Select Master Mix RT-PCR system. *ZmGAPDH* and *ZmACTIN* were used as internal references, and all primers for expression analysis are shown in Table S1 [42,43]. The results of qRT-PCR were calculated using the 2^−ΔΔct^ method, and the expressions of genes in maize samples treated at 22 °C for 3 d under cold stress and at 24 °C for 4 h under heat stress were used as the calibrators [44].

### 2.8. Statistical Analysis

The statistical analysis was conducted by SPSS statistics 22.0, and the Student’s t-test was used to determine the significance levels. The significant level was *p* < 0.05. Data are presented as mean ± SD (standard deviation).

## 3. Results

### 3.1. Characterization of Fatty Acid Desaturase Families in Maize

To identify the whole of FAD families in maize, a genome-wide screening was performed by Blastp using the *Arabidopsis* homologs as query sequences. A total of 17 genes coding for membrane-bound FADs were retrieved, including 13 *ZmFADs*, one *ZmDES*, and three *ZmSLDs*. At the same time, a total of 13 *ZmFAB2s* encoding soluble FAB2s were identified. It is worthwhile to note that there are no evolutionary relationships between the membrane-bound FADs and the soluble FAB2s. The identified *FAD* and *FAB2* genes were named after their counterparts in *Arabidopsis,* and their characteristics are listed on Table S2. The encoded proteins of the membrane-bound FADs ranged from 263 to 556 amino acids, and the predicted molecular weight varied from 28.1 KDa to 93.4 KDa, with the isoelectric points differing from 6.86 to 11.38, respectively (Table S2). The length of encoded soluble FAB2s ranged from 380 to 606 amino acids, the predicted molecular weight varied from 44.4 KDa to 66.7 KDa, and the calculated isoelectric points differed from 6.09 to 9.32 (Table S2).

### 3.2. Phylogenetic Analysis of Maize Fatty Acid Desaturases

**Figure 1:**
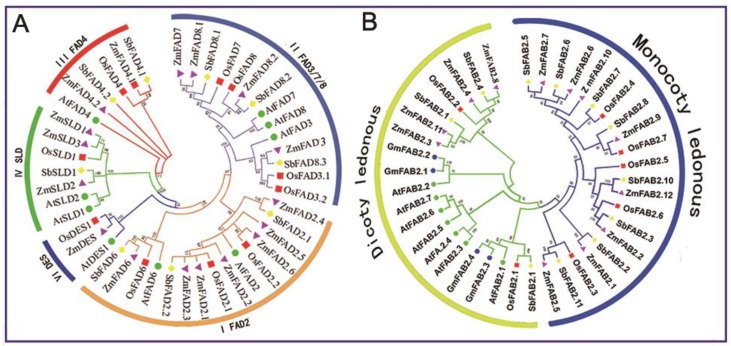
Phylogenetic relationships of fatty acid desaturases (FADs) in different plant species. (**A**) Membrane-bound FADs; (**B**) Soluble FAB2s. The full-length amino acid sequences of FADs from maize (*Z. mays*), *Arabidopsis* (*A. thaliana*), rice (*O. sativa*), sorghum (*S. bicolor*), and soybean (*G. max*) were obtained from an online NCBI database. The phylogenetic trees were constructed using the MEGA 5.0. The detailed information of these genes can be seen in Supplementary Table S3.

The full-length amino acid sequences of FADs and FAB2s from maize (*Z. mays*), *Arabidopsis* (*A. thaliana*), rice (*O. sativa*), sorghum (*S. bicolor*), and soybean (*G. max*) were obtained from an online database (https://www.ncbi.nlm.nih.gov/)using NCBI BLASTp tools. Since there is no evolutionary relationship between the membrane-bound FADs and the soluble FAB2s, a separate phylogenetic tree was constructed for each group (Figure 1).

Figure 1A is the phylogenetic tree of the membrane-bound FADs, which indicated that the membrane-bound FADs could be divided into five sub-groups (the detailed information is listed in Supplementary Table S3). The sub-group I contained the ω-6 desaturases ZmFAD2s, ZmFAD6, and their orthologs from other plants species. The sub-group II was composed of the ω-3 desaturases ZmFAD3, ZmFAD7/8, and their orthologs. The sub-group III harbored two ZmFAD4 and other orthologous proteins, which were *trans* Δ3 desaturases. ZmSLDs and their orthologs fell into sub-group IV, catalyzing the desaturation of sphingolipid at the Δ8 position. The sub-group V contained the sphingolipid Δ4 desaturase ZmDES and its orthologs. Within each sub-group, most maize FADs and their sorghum homologs formed a phylogenetic branch at higher values, reflecting a high degree of sequence homology. Figure 1B shows the phylogenetic tree of the soluble FAB2s (the detailed information is listed in Supplementary Table S3). The FAB2s were divided into two sub-groups—monocotyledonous and dicotyledonous groups. High homology was observed among maize, sorghum, and rice homologs within the monocotyledon sub-group. Similarly, there was a close homologous relationship between *Arabidopsis* and soybean within the dicotyledon sub-group.

### 3.3. Exon/Intron Organization of Maize FAD and FAB2 Genes

**Figure 2:**
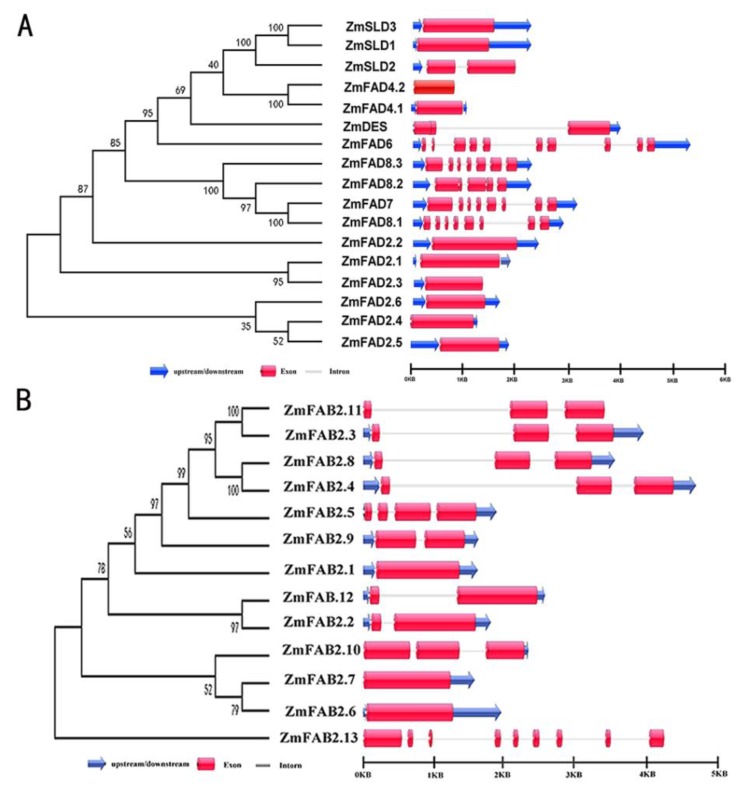
Structure analysis of genes encoding FADs in maize. (**A**) Membrane-bound FADs; (**B**) Soluble FAB2s. The phylogenetic tree is shown on the left, and exon/intron organization is shown on the right. Exons and introns are indicated by red boxes and gray lines, respectively, and untranslated regions are represented by blue arrows.

The structures of maize *FAD* and *FAB2* genes were analyzed using the online analysis software GSDS (http://gsds.cbi.pku.edu.cn/). The configuration analysis revealed that there were big differences in the number of exons/introns. Similar patterns of gene structure were observed in each sub-group. As shown in Figure 2A, most of the genes coding for membrane-bound *FADs* in maize had no intron or only one, except for the plastidial *ZmFAD6/7/8*, which had multiple introns. For most *ZmFAB2s*, the numbers of introns were among zero to two, and only the *ZmFAB2.13* had eight introns (Figure 2B). The similarity of exon/intron structure of maize *FAD* genes implies that they have undergone gene duplication during evolution.

### 3.4. Syntenic Relations of Fatty Acid Desaturase Genes

**Figure 3:**
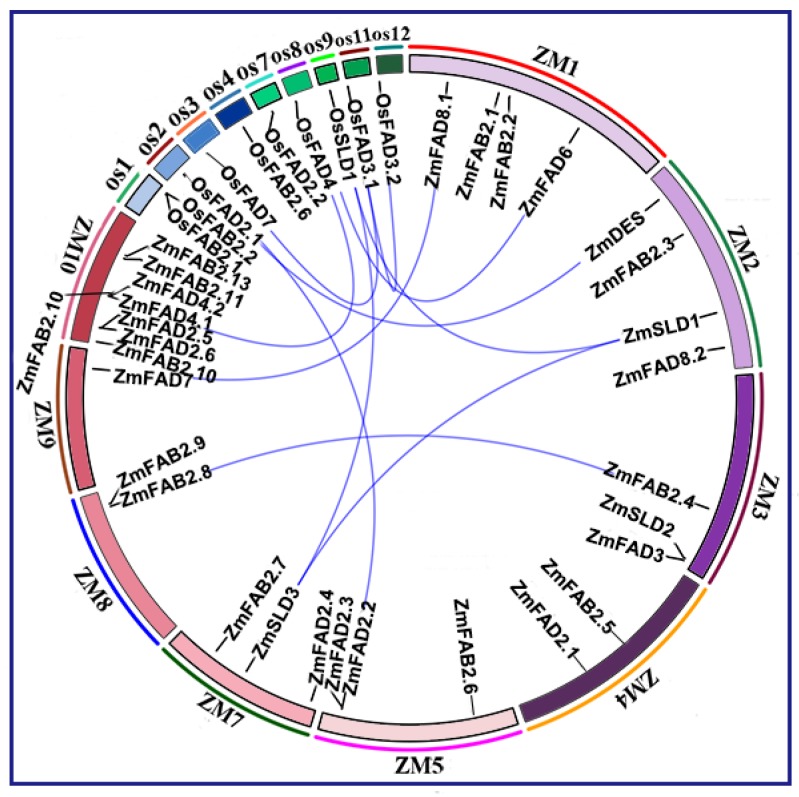
Syntenic analysis of FAD genes from maize and rice. The chromosomes are described as a circle. The colored segments represent the syntenic relationship among the *FAD* and the *FAB2* genes.

The syntenic analysis was conducted on all *FAD* and *FAB2* genes in maize and rice. As shown in Figure 3, the maize *FAD* and *FAB2* genes were mapped on nine out of 10 maize chromosomes (all but chromosome six), and more than half of the genes distributed on chromosome one, two, and 10. There were four genes located in chromosomes one and two, respectively, and a total of eight genes localized in chromosome 10. Only one single gene was found on chromosomes eight and nine, which were *ZmFAB2* and *ZmFAD7*, respectively (Figure 3, Figure S1, and Table S4). We further explored the replication relationship among *FAD* and *FAB2* genes in maize and rice. A total of four duplicated gene pairs (nine duplicated genes) were found, including *OsFAD2.1/ZmFAD2.2, ZmSLD3/ZmSLD1/OsSLD1*, *ZmFAB2.8/ZmFAB2.4*, *ZmFAD7/ZmFAD8.1,* and *ZmFAD4.1/OsFAD4* (Figure 3, Table S4). They aroused mainly through segmental duplication events, and apparently no tandem replication event occurred. The duplicated genes exemplified their common genomic origin and plausible functional similarity.

### 3.5. Conserved Domain Analysis of Maize Fatty Acid Desaturases

**Figure 4:**
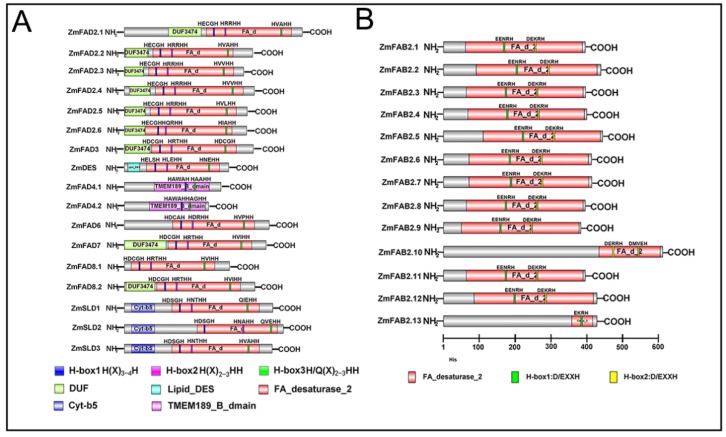
The conserved domains of maize FAD proteins. (A) Membrane-bound FADs; (B) Soluble FAB2s. Predicted signal peptides are shown as colored rectangles. The numbered bar indicates the amino acids.

The conserved domains and the functional motifs of maize FADs were analyzed. As shown in Figure 4A, in the group of membrane-bound FADs, a highly conserved “FA_desatursae domain (PFOO487)” was discovered in almost all FADs, including ZmFADs, ZmDES, and ZmSLDs, which occupied the major part of the proteins. A conserved DUF3474 was present in the N-terminus of most ZmFADs except for ZmFAD4. A distinct “TMEM189_B_dmain domain” was exclusively found in ZmFAD4.1 and ZmFAD4.2, which is a transmembrane domain and may harbor a metal center involved in catalysis [31,45]. A Cyt-b5 (b5-like heme-binding) domain was found at the N-terminus of three ZmSLDs, which might contain the key amino acid site for enzyme function [18]. The ZmDES harbored a “DES-lipids” domain at the N-terminus, which is presumably involved in C4-carboxylation [46]. In most membrane-bound maize FADs, three conserved histidine boxes (histidine rich sequences) were revealed, which might be involved in the formation of enzyme active sites, as has been verified in some divalent iron enzymes [47,48] (Figure 4A). In ZmFAD2s, the three histidine box sequences were HECGH, HRRHH, and HVVHH. However, the ZmFAD4.1 and the ZmFAD4.2 had only two histidine sequences at the N-terminal, while the third histidine box was missing. As shown in Figure 4B, a typical FA_desatursae_2 (PF03405) domain was revealed in all the soluble ZmFAB2s, which was different from that in the membrane-bound FADs and utilized the fatty acyl-ACP as substrates instead of fatty acyl-CoA. Two D/EXXH conserved histidine sequences were also found in the soluble ZmFABs (Figure 4B).

### 3.6. Expression Profiles of Maize Fatty Acid Desaturase Genes in Diverse Tissues

**Figure 5:**
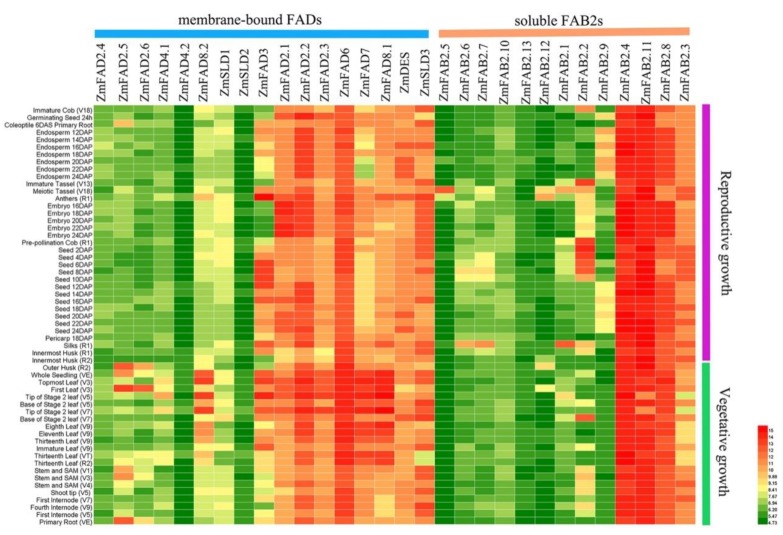
Heat map of expression levels of maize *FAD* and *FAB2* genes in different tissues or at different growth stages. The red boxes indicate high transcript levels, and green boxes indicate low transcript levels.

To determine the expression profiles of maize FAD genes in different tissues and developmental stages, we searched the Maize eFP database and performed the expression analysis. As demonstrated in Figure 5, the expression of FAD genes varied in different tissues. In the group of *FADs*, *ZmFAD2.1*–*2.3* exhibited high expression levels in various tissues at both vegetative and reproductive growth stages; however, *ZmFAD2.4*–*2.6* exhibited lower expression levels. These observations indicate that some of the *ZmFAD2s* might play important roles in maize growth and development. In the reproductive growth stages, *ZmFAD3* exhibited higher expression levels during seed development, suggesting its possible involvement in seed development. In the vegetative growth stages, *ZmFAD6/7/8* possessed higher expression levels in different stages of leaves, indicative of vital functions in chloroplast and photosynthesis. In the group of *FAB2s*, *ZmFAB2.3*, *ZmFAB2.4*, *ZmFAB2.8*, and *ZmFAB2.11* showed higher expression levels than other *ZmFAB2* genes in tissues at both vegetative and reproductive growth stages.

### 3.7. Expression Profiles of the Maize Fatty Acid Desaturase Genes in Response to Temperature Stresses

**Figure 6:**
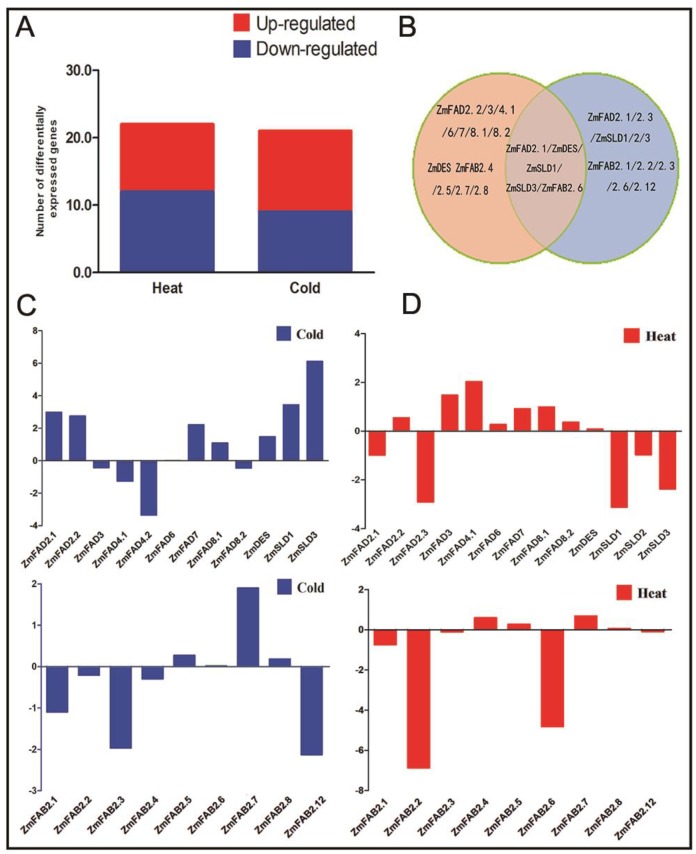
Expression profiles of the maize *FAD* and *FAB2* genes under cold and heat stresses. (**A**) The number of up/down-regulated genes under cold and heat stresses; (**B**) Venn diagram describes the overlapping genes reacting on heat and cold stress; (**C**) expression of genes under cold stress; (**D**) expression of genes under heat stress.

To explore the responses of maize *FAD* and *FAB2* genes under temperature stresses, we examined their expression patterns under cold or heat stresses. The transcriptome data of maize under cold stress were obtained from our previous study (SRX2672484) [39], while the transcriptome data of maize under heat stress were downloaded from the NCBI sequence-reading archive (SAR) (SRR1238715, SRR1819196, and SRR1819198) [40]. As shown in Figure 6A, these *FAD* and *FAB2* genes displayed differential expression profiles against cold and heat stresses. Under cold stress, the expression of 12 genes was up-regulated, and for nine genes, it was down-regulated. Under heat stress, the expression of 12 *FAD* genes was up-regulated and expression was down-regulated for 10 genes. Notably, 30 of these *ZmFAD* and *FAB2* genes showed differential expression under cold and heat stresses (Figure 6B). Among them, *ZmFAD2.1*, *ZmDES*, *ZmSLD1/3,* and *ZmFAB2.6* were up-regulated under cold stress and down-regulated under heat stress, respectively. The expression profiles of maize *FAD* and *FAB2* genes are illustrated in Figure 6C,D. Under cold stress, the expression of *ZmFAD2.1, ZmFAD2.2*, and *ZmSLD1–3* genes was significantly up-regulated. The expression of *ZmFAB2.7* was up-regulated under cold stress, whereas the expression of *ZmFAB2.1*, *ZmFAB2.3*, and *ZmFAB2.12* was down-regulated (Figure 6C). Under heat stress, an obvious down-regulation of *ZmFAD2.1*, *ZmFAD2.3,* and *ZmSLD1–3* genes was observed, which was opposite to their responses to cold stress. The expression of *ZmFAB2.2* and *ZmFAB2.6* was significantly down-regulated under heat (Figure 6D). These results reflect the need of increased fatty acid unsaturation for plants to acclimate to cold stress and reduced fatty acid unsaturation for plants to cope with heat stress.

### 3.8. Co-Expression Analysis of Transcription Factors and ZmFADs in Maize

**Figure 7:**
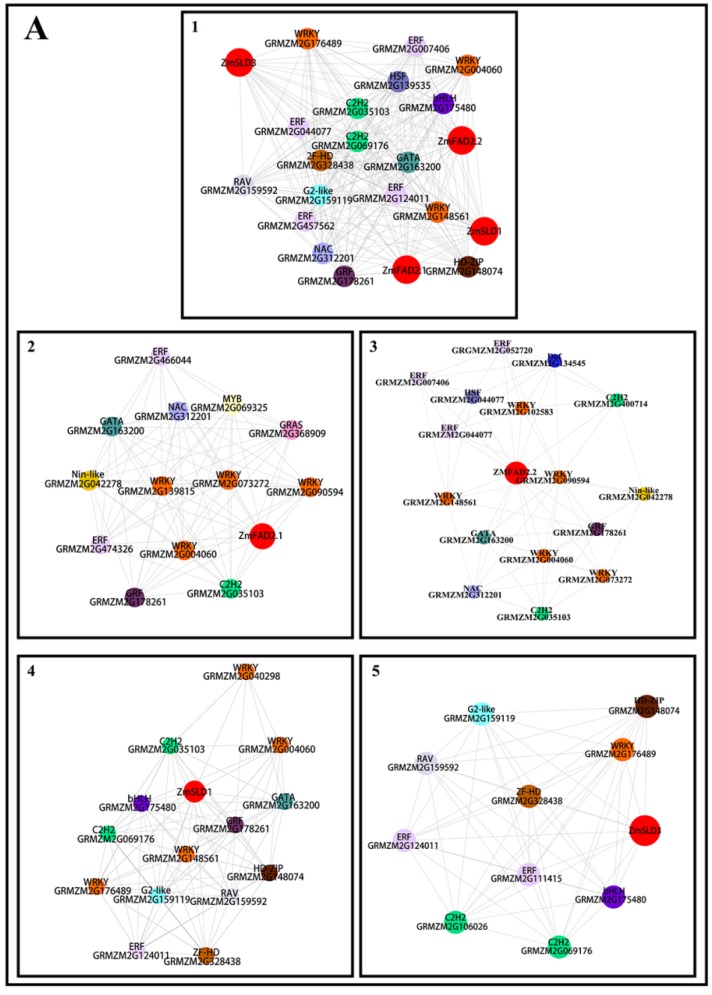

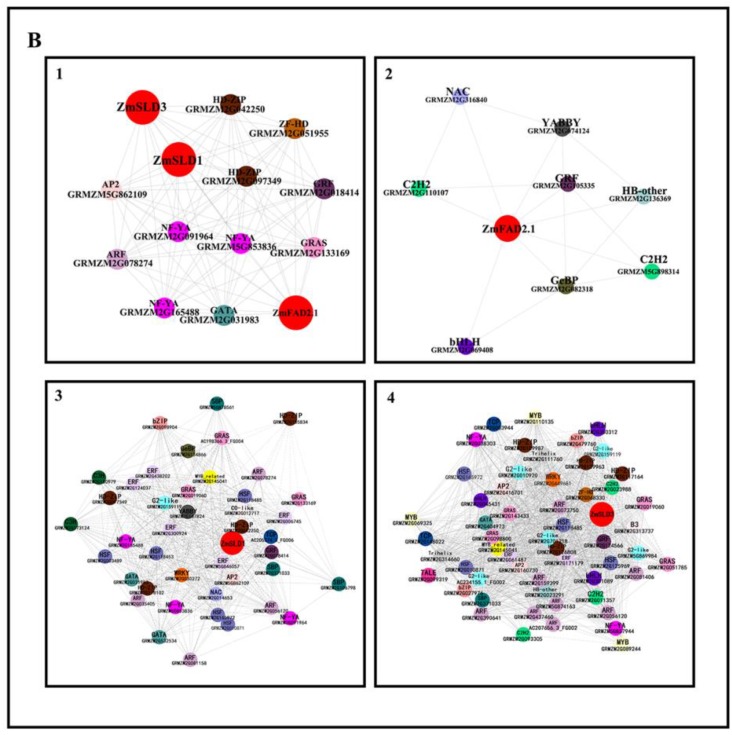
Co-expression network of transcription factors and maize *FADs*. (**A**) Under cold stress; (**B**) Under heat stress. The co-expression relationship was established when the Pearson correlation was higher than 0.97, and the co-expression network was built using the Perl script with a default value of 0.6. Different colored circles represent different transcription factors.

The significant response of a number of maize *FAD* genes prompted us to explore their regulation at the transcriptional level. The co-expression analysis of transcription factors (TFs) and the high responsive *FADs* was conducted using Cytoscape v 3.4.10. The transcription factors were screened from the above mentioned cold or heat transcriptome data using plant TF database PlanTFDB (http://planttfdb.cbi.pku.edu.cn/). In the cold transcriptome of maize, around 1000 transcription factors were identified, among which the most abundant TF families were ERF, WRKY, C2H2, and bHLH. The strongly cold-responsive genes *ZmFAD2.1*, *ZmFAD2.2*, *ZmSLD1,* and *ZmSLD3* were named “guide genes” and were used as the inquiry to execute the co-expression relationships with corresponding transcription factors. As shown in Figure 7 a total of 18 transcription factors were shown to be involved in the regulation of all four guide genes, including ERF(5), WRKY(4), C2HC(2), and bHLH(1), and a co-expression network was established among the guide genes and the corresponding TFs (Figure 7A1, Table S5).

The co-expression networks were also generated for individual guide gene. Five transcription factors were found to be significantly correlated with the expression of *ZmFAD2.1*, including ERF (GRMZM2G474326, r = 0.993), GATA (GRMZM2G163200, r = 0.995), MYB (GRMZM2G069325, r = 0.993), WRKY (GRMZM2G073272, r = 0.994), and WRKY (GRMZM2G139815, 0.994) (Figure 7A2). Eight transcription factors were markedly correlated with *ZmFAD2.2*, including WRKY (GRMZM2G004060, r = 0.993), WRKY (GRMZM2G090594, r = 0.998), WRKY (GRMZM2G102583, r = 0.995), WRKY (GRMZM2G148561, r = 0.995), ERF (GRMZM2G044077, r = 0.993), ERF (GRMZM2G052720, r = 0.992), NAC (GRMZM2G312201, r = 0.994), Dof (GRMZM2G134545, r= 0.996), and WRKY (GRMZM2G073272) [Figure 7A3]. Thus, these TFs may mainly regulate the response of *ZmFAD2.1* to low temperature stress. HD-ZIP (GRMZM2G148074, r = 0.984), GRF (GRMZM2G178261, r = 0.983), RAV (GRMZM2G159592, r = 0.981), C2H2 (GRMZM2G035103, r = 0.979), bHLH (GRMZM2G175480, r = 0.978) and (GRMZM2G106026, r = 0.995), HD-ZIP (GRMZM2G148074, r = 0.995), bHLH (GRMZM2G175480, r = 0.993), ERF (GRMZM2G124011, r = 0.992), and WRKY (GRMZM2G176489, r = 0.991) were predicted to be correlated with *ZmSLD1* and *ZmSLD3*, respectively (Figure 7A4,A5).

In the heat transcriptome of maize, 377 transcription factors were identified, among which the most abundant TF families were ERF, bHLH, and MYB. Since the expression of *ZmFAD2.1*, *ZmSLD1,* and *ZmSLD3* were found to be significantly down-regulated under heat stress, they were used as the “guide genes” to study the co-expression relationships with transcription factors. A total of 11 TFs exhibited co-expression relationships with all three guide genes, including NF-YA (3), HD-ZIP (2), AP2 (1), ARF (1), GATA (1), GRAS (1), GRF (1), and ZF-HD (1) (Figure 7B1). Five transcription factors were discovered to be significantly correlated with *ZmFAD2.1*, including C2H2 (GRMZM2G110107, r = 0.993), NAC (GRMZM2G316840, r = 0.993), YABBY (GRMZM2G074124, r = 0.99), GRF (GRMZM2G105335, r = 0.985), and C2H2 (GRMZM5G898314, r = 0.985) (Figure 7B2, Table S5). A total of 42 transcription factors were identified, displaying a significant correlation with the expression of *ZmSLD1* in both positive and negative directions. The positively correlated TFs were four ARFs, three NF-YAs, and three SBPs (r > 0.99) (Figure 7B3). The five negatively correlated TFs were ERF and HSF (r < −0.99). Fifty-four TFs had significant correction with *ZmSLD3*, and eight dominant TFs were ARF (eight), bHLH (three), C2H2 (three), G2-like (five), GRAS (four), HD-ZIP (four), HSF (three), and MYB (three) (Figure 7B4). 

### 3.9. The qRT-PCR Analysis of the Expression of ZmFAD and ZmTF Genes under Cold and Heat Stresses

**Figure 8:**
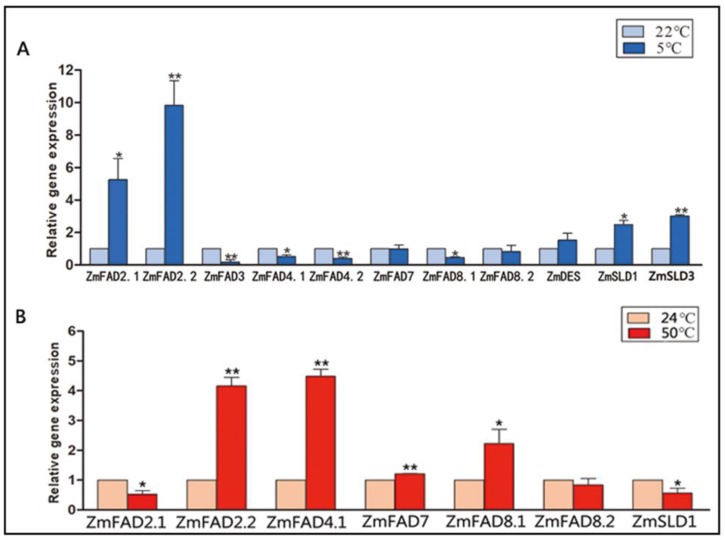
The qRT-PCR analysis of the expression profiles of *ZmFADs* in response to cold (**A**) and heat; (**B**) treatment in maize leaves.

In order to validate the gene expression in response to temperature stress in this study, qRT-PCR analysis was conducted on a number of ZmFAD/SLD genes (FAD2.1, 2.2, and SLD1, 3) and four TFs, which were selected from the co-expression network based on their high correlation with the ZmFAD/SLD genes. Under cold stress (Figure 8A), the expression profiles of ZmFAD/SLD genes determined by qRT-PCR were highly consistent with those generated from the transcriptome data, as shown in Figure 6C (R = 0.7180, Figure S2). Under heat stress (Figure 8B), the expression profiles of ZmFAD/SLD genes and the previous results were also highly correlated (R = 0.9699, Figure 8B, Figure S3). The qRT-PCR analysis of the expression of the genes encoding the four TFs revealed their expression profiles were significantly correlated with those of FAD2.1, SLD1 and SLD3, which evidenced the close association predicted in the above co-expression network (Figure (S4,S5)).

## 4. Discussion

**Figure 9:**
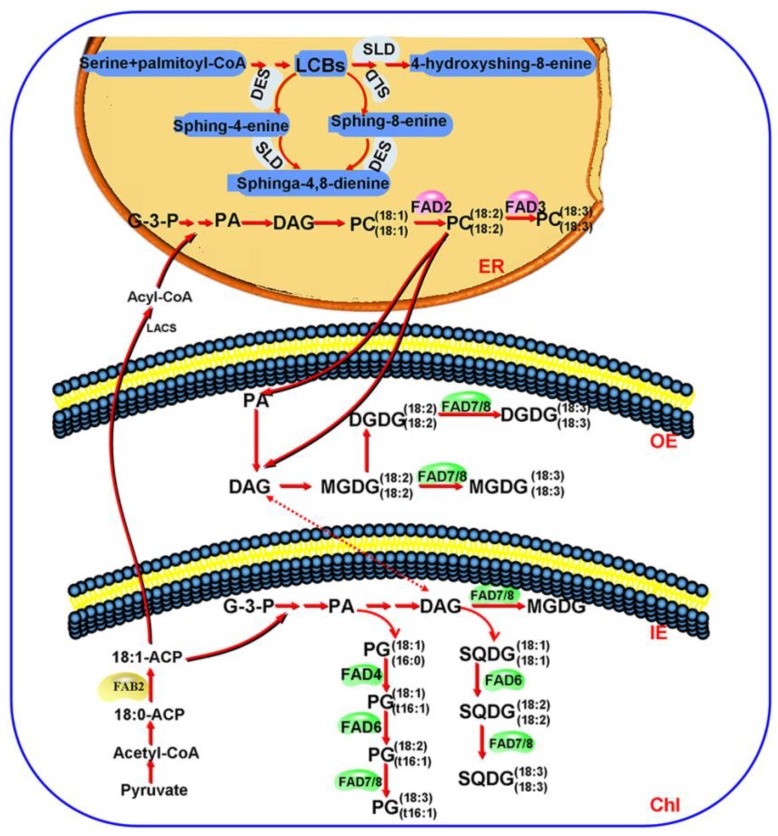
Diagram presenting the roles of fatty acid desaturases in the lipid metabolism of maize. ER: endoplasmic reticulum; OE: outer envelope; IE: inner envelope; Chl: chloroplast; G-3-P: glycerol triphosphate; PA: phosphatidic acid; DAG: diacylglycerol; PC: phosphatidylcholine; LCB: long-chain base; PG: phosphatidyl glycerol; DGDG: digalactosyldiacylglycerol; MGDG: monogalactosyldiacylglycerol; SQDG: sulfoquinovosyl diacylglycerol; LCB: long-chain bases; DES: sphingolipid Δ4-desaturases; SLD: sphingoid LCB Δ8 desaturase; FAD: membrane-bound fatty acid desaturases; FAB2: soluble fatty acid desaturases.

As the key factor in determining the content of unsaturated fatty acids in plants, FADs play important roles in plant growth and respond to various stresses [4,28]. Not much information is available on the comprehensive FADs families in maize, although *FAD* genes from a range of plant species have been identified and studied [6,49]. In this study, we identified a total of 30 genes encoding different types of FADs from the maize genome. Their possible participation in the regulation of fatty acid desaturation is illustrated in Figure 9.

In this study, genes encoding the membrane-bound ZmFAD2-3, ZmFAD4, and ZmFAD6-8 were identified from online databases using the *Arabidopsis* homologous genes as searching queries, except the gene coding for ZmFAD5, which was not found from maize genome. As we mentioned earlier, there are two spatially independent pathways for the synthesis of glycerolipids in plants—the ER localized eukaryotic pathway and the chloroplast localized prokaryotic pathway [1]. Some plants, such as *Arabidopsis*, whose major chloroplast lipids [monogalactosyldiacylglycerol (MGDG) and digalactosyldiacylglycerol (DGDG)] are generated jointly by both pathways, are typically characterized by high levels of 16:3 fatty acids and are termed 16:3 plants [50,51]. In others such as maize, their MGDG and DGDG synthesis almost entirely relies on the ER pathway, and the products have high levels of 18:3 fatty acids, thus they are called 18:3 plants [1,52]. In the 18:3 plants maize, the synthesis of galactolipids (MGDG and DGDG) relies on the ER-localized eukaryotic pathway, whereas the chloroplast-localized prokaryotic pathway is not functioning [1,39]. FAD5 catalyzes the early step of MGDG desaturation in the chloroplast-localized prokaryotic pathway in *Arabidopsis*, which is a 16:3 plants that uses both the eukaryotic and the prokaryotic pathways for galactolipid synthesis [1,39]. Hence, the dysfunction of the chloroplast prokaryotic pathway in the 18:3 maize plants might be one reason for the absence of a gene coding for ZmFAD5 (Figure 9).

Phylogenetic analysis provided clear evidence for the lack of evolutionary relationships between soluble and membrane-bound FADs in maize. In the membrane-bound FADs group, 17 membrane-bound FADs were classified into five sub-groups based on their phylogenetic relationships. Since FADs possess unique substrate specificities, such different FAD types mediate the desaturation reactions on lipids/acyl-ACPs being localized in different subcellular localizations and at distinct positions on the FA chains [14]. The phylogenetic classification of maize FADs featured their specific functional characteristics. The sub-group I harbored the ω-6 desaturases ZmFAD2s, ZmFAD6, and their orthologs from other plants species, which catalyzed the double bond insertion in the ω-6/Δ-12 position; however, FAD2 and FAD6 were localized to the ER and to the plastid, respectively [45]. The sub-group II was composed of the ω-3 desaturases ZmFAD3 and ZmFAD7/8, which converted linoleate (18:2) substrates esterified to PC or plastidial lipids to linolenate (18:3) lipid species [45]. The sub-group III contained two ZmFAD4, which were *trans* Δ3 desaturases introducing a *trans* double bond into PG (16:0, 18:1) to form PG (t16:1, 18:1) [16,20]. The sub-group IV was SLDs, which catalyzed the desaturation of sphingolipid at the Δ8 position, whereas the sphingolipid Δ4 desaturase ZmDES in sub-group V inserted a *trans* double bond between the Δ4 and the Δ5 position [18,20].

Although there is no evolutionary relationship between maize soluble and membrane-bound FADs, they all had a highly conserved FA_desaturase domain, and the types and the distribution of the conserved motifs were similar within each sub-group. The maize membrane-bound FADs also have a number of unique protein domains, such as TMEM189_B_Dmain, b5-like hem-binding, and DES-lipids domains, which may be essential for their functions. In a previous study, it was reported that the cotton FAD contains conserved histidine boxes (five to six amino acid sequences), which might be involved in the formation of enzyme active sites, as has been verified in some divalent iron enzymes [47]. In addition, three conserved histidine boxes (histidine rich sequences) were revealed in the membrane-bound FADs. Similarly, we found that membrane-bound FADs in maize also contained three conserved histidine boxes, except ZmFAD4.1 and ZmFAD4.2, which had two histidine sequences in the N-terminal, while the third histidine box was missing. Soluble FAB2s are localized to plastids and are thus exclusively found in plants; they contain two conserved histidine sequences D/EXXH [53]. In agreement with this, we found that soluble FAB2s in maize also harbored two conserved histidine boxes.

In this study, to explore the response of maize *FADs* to temperature stresses, we examined the expression pattern of the *FAD* genes via transcriptome data analysis. The results showed that the expression of *ZmFAD2.1-2.2* and *ZmSLD1-3* was significantly up-regulated under cold stress, whereas under heat stress, obvious down-regulation of *ZmFAD2.1*, *ZmFAD2.3,* and *ZmSLD1*-3 was observed. A recent publication reviewed that different expression patterns of plant *FAD2* are associated with content of unsaturated fatty acids, indicating *FAD2* genes have direct effects on plasma membrane adaptability under cold stress [54]. It has been reported in a number of plants that when FAD2 dysfunctions, the fatty acid unsaturation decreases and the cold resistance of plants weakens [55,56]. Yeast strains transformed with sunflower *FAD2-1* and *FAD2-3* genes show a high degree of unsaturated lipids, higher membrane lipid fluidity, and improved low temperature and salt tolerance [57]. The *Arabidopsis* mutant deficient in *SLD* gene displays increased sensitivity to low temperature [18]. In tomato, silencing of *SlSLD* results in the accumulation of higher levels of saturated LCB, which causes severe apoptosis under low temperature stress [58]. In the eukaryotic pathway, FAD2 participates in the desaturation of PC (18:1) to form PC (18:2), which could be degraded to provide substrate for the biosynthesis of plastidic lipids (MGDG and DGDG) [54]. In maize studied herein, under low temperatures, the expression of *ZmFAD2* was obviously induced, and the PC (18:2) content was increased, which further improved MDGD and DGDG biosynthesis in chloroplasts [39]. The responses of maize *FAD* and *SLD* genes to cold and heat suggest their plausible roles in the regulation of fatty acid desaturation and plasma/photosynthetic membrane modification and maintaining the stability of cell membranes under temperature stresses.

It is well known that transcription factors participate in the regulation of gene expression under various abiotic stress; however, the correlation of transcription factors and *FAD* genes has not been well established yet [59]. In this study, the co-expression analysis of transcription factors and the most responsive *FADs* was conducted, and we found that expressions of genes coding for WRKY, ERF, C2H2, and bHLH TF classes displayed a significant connection with those of *ZmFAD2.1*/*2.2* and *ZmSLD1*/*3* under cold stress, while HD-ZIP, NY-YA, and HSF apparently correlated with the expression of target *FAD* genes under heat stress conditions. The *cis-*regulatory elements associated with some TFs and cold stress responses were predicted in the promoter regions of maize *FAD* and *FAB2* genes (Figure S6), which revealed a number of various *cis-*elements in each gene and the feasible regulation of maize *FAD* genes at the transcriptional level. This co-expression analysis of transcription factors and maize *FAD* genes provides the primary information on understanding the regulation of *FADs* in response to temperature stresses, but further investigation will be needed to decipher the transcriptional regulation network.

## Supplementary Materials

The following are available online at https://www.mdpi.com/2073-4425/10/6/445/s1. Figure S1: Chromosome location of fatty acid desaturase gene families; Figure S2: Correlations of expression levels with *FAD* genes under cold stress; Figure S3: Correlations of expression levels with *FAD* genes under heat stress; Figure S4: Expression level of transcription factors under cold stress and correlations of expression levels between *FAD2.1–2.2*, *SLD1/3* genes and transcription factors under cold stress; Figure S5: Expression level of transcription factors under heat stress and correlations of expression levels between *FAD2.1*, *SLD1/3* genes and transcription factors under heat stress; Figure S6: The *cis-*regulatory elements associated with some TFs and cold stress responses were predicted in the promoter regions of maize *FAD* genes; Table S1: Primers used for qRT-PCR; Table S2: Characteristics of fatty acid desaturase from maize; Table S3: The gene ID of fatty acid desaturase genes used in this study; Table S4: The syntenic relationships among maize and rice fatty acid desaturase genes; Table S5: The Pearson correlation analysis between TFs and target fatty acid desaturase genes.
