# Supplementary figures and images for: Identification of Fatty Acid Desaturases in Maize and Their Differential Responses to Low and High Temperature
Source: Genes (Basel). 2019 Jun 12;10(6):445. doi: 10.3390/genes10060445 (PMC6627218; doi:10.3390/genes10060445)

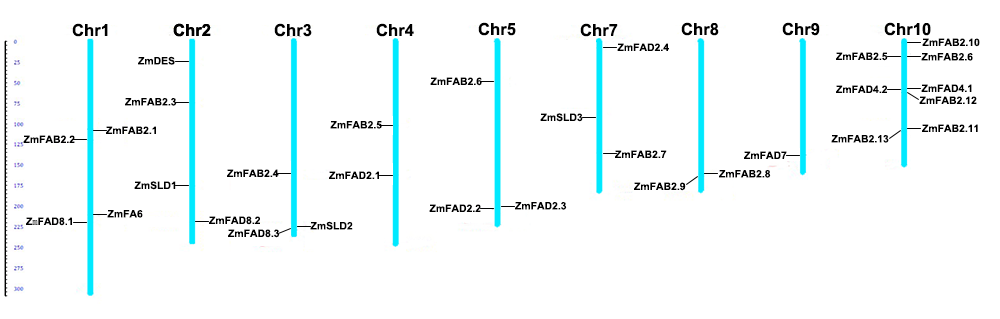

Supplement: Supplementary file 1 [file genes-10-00445-s001.zip › Supplementary Figures and Tables/FigureS1.tif]

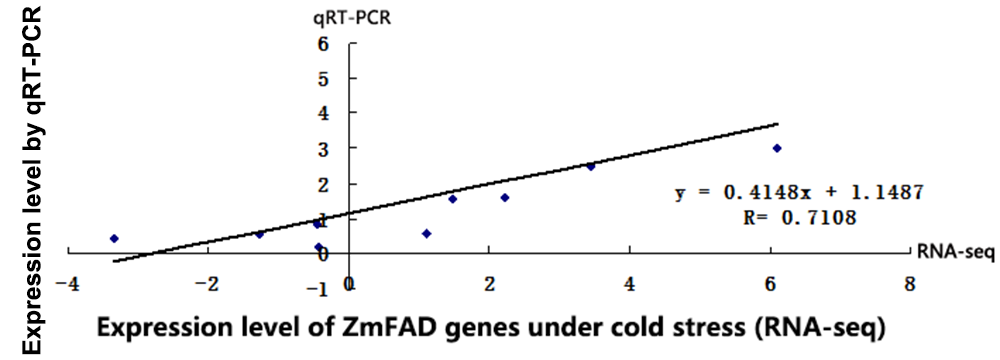

Supplement: Supplementary file 1 [file genes-10-00445-s001.zip › Supplementary Figures and Tables/FigureS2.tif]

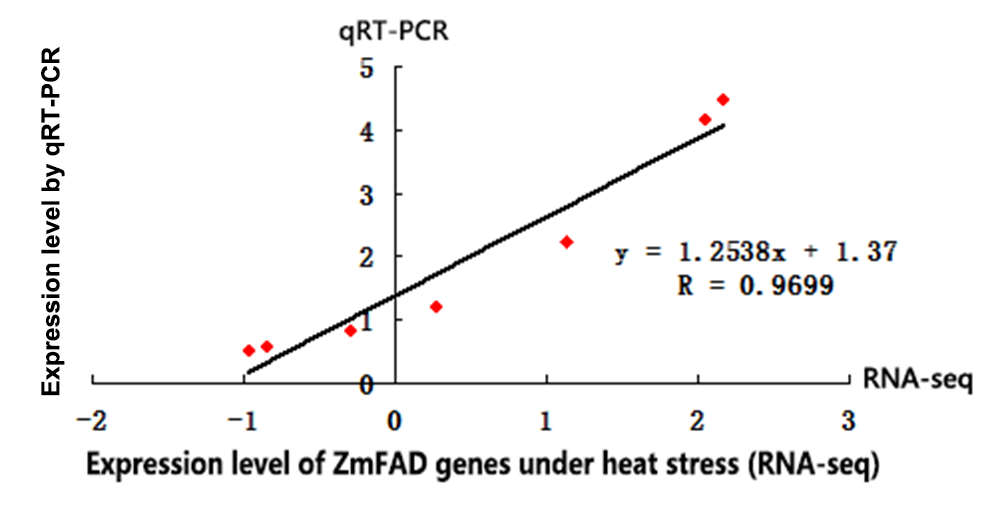

Supplement: Supplementary file 1 [file genes-10-00445-s001.zip › Supplementary Figures and Tables/FigureS3.tif]

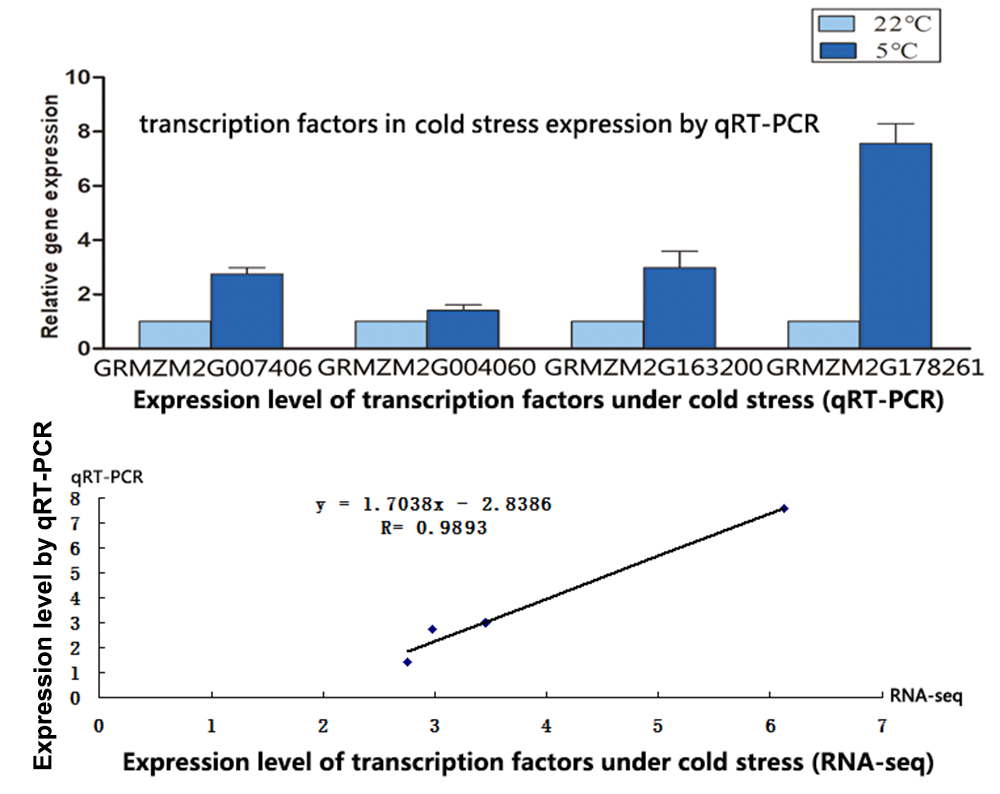

Supplement: Supplementary file 1 [file genes-10-00445-s001.zip › Supplementary Figures and Tables/FigureS4 .tif]

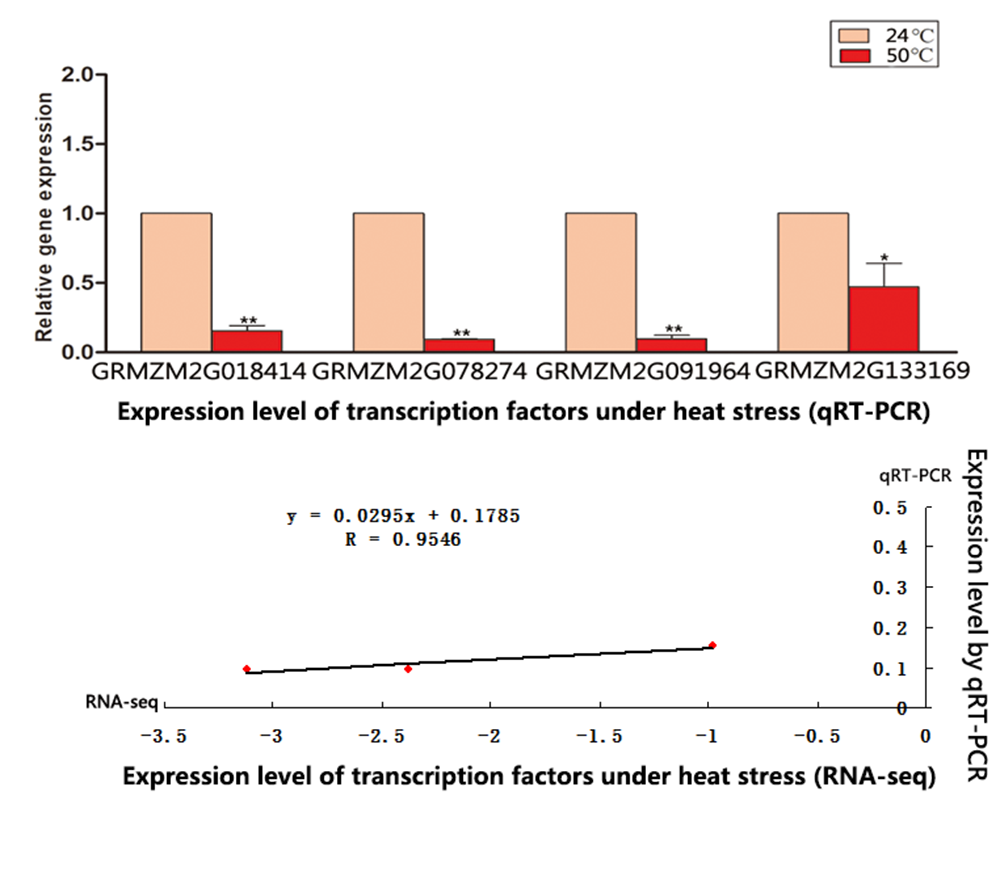

Supplement: Supplementary file 1 [file genes-10-00445-s001.zip › Supplementary Figures and Tables/FigureS5.tif]
