# Supplementary material for: Identification of Fatty Acid Desaturases in Maize and Their Differential Responses to Low and High Temperature
Source: Genes (Basel). 2019 Jun 12;10(6):445. doi: 10.3390/genes10060445 (PMC6627218; doi:10.3390/genes10060445)
Supplement: Supplementary file 1 [file genes-10-00445-s001.zip › Supplementary Figures and Tables/Table S1.doc]

**Supplemental Table S1 Primers used for quantitive real-time PCR**

Fatty acid desaturase gene primer used under heat and cold stress

Primer Names	Sequence (5'-3')

ZmACTIN-qF	ATCCAGGCTGTTCTTTCGTT

ZmACTIN-qR	CATTAGGTGGTCGGTGAGGT

ZmGAPDH-qF	CCCTTCATCACCACGGACTAC

ZmGAPDH-qR	AACCTTCTTGGCACCACCCT

ZmFAD2.1-qF	ACCACCTCTTCTCCACCATGCC

ZmFAD2.1-qR	CCTTGCGGTTGCGGTTCTCAG

ZmFAD2.2-qF	TGCCGCTGCTGATCGTGAAC

ZmFAD2.2-qR	TGCGTGTCCGTGATGTTGTGG

ZmFAD3-qF	GGACTACGGCTGGATCAACAACG

ZmFAD3-qR	ACGACGAGGAGGTGAAGTGGAAG

ZmFAD4.1qF	CACCGCCAGCCGTACAACAC

ZmFAD4.1qR	CGCCTCGAACACCTTGTATCTGTC

ZmFAD4.2qF	CACCATCGTCCGCCGTACAAC

ZmFAD4.2qR	CCGTGCTCCGTCATCTTCATTCG

ZmFAD7-qF	TGTTGCTTGGCTGGATATGGTCAC

ZmFAD7- qR	TCCTCCACGCAGATAACTCCACTC

ZmFAD8.1-qF	GGTTGGTGTTCTGGCTGGTCTC

ZmFAD8.1-qR	CCTCCACGCAGATAACTCCATTCC

ZmFAD8.2-qF	CACAGGACGCACCACCAGAAC

ZmFAD8.2-qR	GACGCTGTCCAGGCTCTTGAAC

ZmSLD1-qF	GGTGGCGTTCGTGCTTCTCAG

ZmSLD1-qR	GTGCCTGCCGTCTGCTTCTC

ZmSLD3-qF	GGTCTCATTGGCTTCGTCTGGATC

ZmSLD3-qR	AGGCGATGTGGTGCGTGTTG

ZmDES-qF	TGGGCCTTCCTCAAGATTGG

ZmDES-qR	AAGGCAAGGTTGTGGCTCA

Transcription factor gene primer used under cold stress

| Primer Names | Sequence (5'-3') |
| --- | --- |
| GRMZM2G007406-qF | TACGACGCCGCTCTGCTCTG |
| GRMZM2G007406-qR | CGGACGTGGAGGAGGATGAGG |
| GRMZM2G004060-qF | GGTTGTGACGAAGAGCGTGAGG |
| GRMZM2G004060-qR | GGTGCTTGCAGTTCTGGATCTCC |
| GRMZM2G163200-qF | GCCTCTTCTGACGACAACGATGG |
| GRMZM2G163200-qR | GGCGTCTTCTCGATCTGGCAATG |
| GRMZM2G178261-qF | CCTGCAACACTTCGTGGACTGG |
| GRMZM2G178261-qR | CTTCAGAGGCGACAGCATCAGC |
|  |  |

Transcription factor gene primer used under heat stress

| Primer Names | Sequence (5'-3') |
| --- | --- |
| GRMZM2G018414-qF | TCGCAGTGGATGGAGCTGGAG |
| GRMZM2G018414-qR | ACGAGCAGGCTGGAAGGTATGG |
| GRMZM2G078274-qF | TTGCTTGGAATCAGACACGCTACC |
| GRMZM2G078274-qR | CTGCTGCAAGGAGTCCGATGTG |
| GRMZM2G091964-qF | AGAACCATGCCACATCGCAGATAC |
| GRMZM2G091964-qR | GAGCCACAGCATGACTTCCATAGG |
| GRMZM2G133169-qF | TGAGCCTGACGAGACTGTAGCC |
| GRMZM2G133169-qR | TGACTTAACCAGCCGGAGGACTC |
