# Supplementary material for: Identification of Fatty Acid Desaturases in Maize and Their Differential Responses to Low and High Temperature
Source: Genes (Basel). 2019 Jun 12;10(6):445. doi: 10.3390/genes10060445 (PMC6627218; doi:10.3390/genes10060445)
Supplement: Supplementary file 1 [file genes-10-00445-s001.zip › Supplementary Figures and Tables/Table S2 .doc]

**TableS2. Characteristics of fatty acid desaturase from maize**

|  | | | **Protein** | | | |
| --- | --- | --- | --- | --- | --- | --- |
| **Gene Name** | **Gene ID^a^** | **ORF^b^**  **length**  **(bp)** | **Length(aa)** | **Molecular weight**  **(KDa)** | **PI^C^** | **Hydrophilic relationship** |
| *ZmFAB2.1* | GRMZM2G143625_T01 | 1179 | 392 | 44.4 | 8.49 | -0.352 |
| *ZmFAB2.2* | GRMZM2G003368_T02 | 1305 | 434 | 48.1 | 8.77 | -0.273 |
| *ZmFAB2.3* | GRMZM2G026793_T01 | 1179 | 392 | 44.7 | 7.13 | -0.477 |
| *ZmFAB2.4* | GRMZM5G852502_T01 | 1191 | 396 | 45.1 | 6.85 | -0.441 |
| *ZmFAB2.5* | GRMZM2G073540_T01 | 1323 | 440 | 4.96 | 7.15 | -0.428 |
| *ZmFAB2.6* | GRMZM2G073540_T01 | 1227 | 408 | 45.4 | 6.4 | -0.251 |
| *ZmFAB2.7* | GRMZM5G883417_T01 | 1230 | 409 | 45.2 | 7.25 | -0.304 |
| *ZmFAB2.8* | GRMZM2G180399_T01 | 1179 | 392 | 44.5 | 6.09 | -0.395 |
| *ZmFAB2.9* | GRMZM2G316362_T01 | 1143 | 380 | 42.4 | 6.58 | -0.34 |
| *ZmFAB2.10* | GRMZM2G317287_T01 | 1821 | 606 | 66.7 | 8.32 | -0.156 |
| *ZmFAB2.11* | AC215690.3_FGT002 | 1179 | 392 | 44.7 | 6.34 | -0.47 |
| *ZmFAB2.12* | GRMZM2G119305_T01 | 1275 | 424 | 47.5 | 8.17 | -0.352 |
| *ZmFAB2.13* | GRMZM2G166956_T01 | 1275 | 424 | 47.1 | 9.32 | -0.424 |
| *ZmFAD2.1* | GRMZM2G064701_T01 | 1671 | 556 | 62 | 9.61 | -0.22 |
| *ZmFAD2.2* | GRMZM2G056252_T04 | 1203 | 400 | 43.8 | 11.3 | -0.421 |
| *ZmFAD2.3* | GRMZM2G161792_T01 | 1122 | 373 | 42.7 | 8.54 | 0.013 |
| *ZmFAD2.4* | GRMZM2G174766_T01 | 1218 | 405 | 44 | 9.04 | 0.072 |
| *ZmFAD2.5* | GRMZM2G169240_T01 | 1155 | 384 | 43.5 | 8.72 | 0.067 |
| *ZmFAD2.6* | GRMZM2G169261_T01 | 1149 | 382 | 43.6 | 8.91 | -0.024 |
| *ZmFAD3* | GRMZM2G354558_T01 | 1209 | 402 | 46.1 | 8.23 | -0.291 |
| *ZmFAD4.1* | GRMZM2G175401_T01 | 909 | 302 | 31.9 | 6.86 | -0.081 |
| *ZmFAD4.2* | GRMZM2G097509_T01 | 792 | 263 | 28.1 | 7.58 | 0.268 |
| *ZmFAD6* | GRMZM2G078569_T04 | 1359 | 452 | 52.2 | 9.24 | -0.113 |
| *ZmFAD7* | GRMZM2G128971_T01 | 1332 | 443 | 93.4 | 9.16 | -0.176 |
| *ZmFAD8.1* | GRMZM2G074401_T01 | 987 | 328 | 34.7 | 8.44 | -0.17 |
| *ZmFAD8.2* | GRMZM2G129209_T01 | 1224 | 407 | 45.9 | 9 | -0.046 |
| *ZmDES* | GRMZM2G078373_T01 | 987 | 325 | 37.2 | 7.89 | -0.07 |
| *ZmSLD1* | GRMZM2G179120_T01 | 1395 | 464 | 51.3 | 8.23 | 0.139 |
| *ZmSLD2* | GRMZM2G430729_T01 | 1491 | 496 | 55.4 | 9.17 | -0.014 |
| *ZmSLD3* | GRMZM2G129453_T01 | 1389 | 462 | 51.3 | 8.48 | 0.142 |

^a^IDs are available in the maize genome sequencing project database ([http://www.maizesequence.org](http://www.maizesequence.org/))

^b^ORF: open reading frame.

^C^PI: Isoelectric point
