# Supplementary material for: Identification of Fatty Acid Desaturases in Maize and Their Differential Responses to Low and High Temperature
Source: Genes (Basel). 2019 Jun 12;10(6):445. doi: 10.3390/genes10060445 (PMC6627218; doi:10.3390/genes10060445)
Supplement: Supplementary file 1 [file genes-10-00445-s001.zip › Supplementary Figures and Tables/Table S3.doc]

**Table S3. The gene ID of fatty acid desaturase genes used in this study**

| **Species** | **Gene locus** | **Gene symbol** |
| --- | --- | --- |
| *Arabidopsis thaliana* | AT3G12120 | AtFAD2 |
|  | AT2G29980 | AtFAD3 |
|  | AT4G27030 | AtFAD4 |
|  | AT4G30950 | AtFAD6 |
|  | AT3G11170 | AtFAD7 |
|  | AT5G05580 | AtFAD8 |
|  | AT4G04930 | AtDES1 |
|  | AT3G61580 | AtSLD1 |
|  | AT2G46210 | AtSLD2 |
| *Oryza sativa* | LOC_Os02g48560 | OsFAD2.1 |
|  | LOC_Os07g23410 | OsFAD2.2 |
|  | LOC_Os07g23430 | OsFAD2.3 |
|  | LOC_Os11g01340 | OsFAD3.1 |
|  | LOC_Os12g01370 | OsFAD3.2 |
|  | LOC_Os08g08850 | OsFAD4 |
|  | LOC_Os08g34220 | OsFAD6 |
|  | LOC_Os03g18070 | OsFAD7 |
|  | LOC_Os07g49310 | OsFAD8 |
|  | LOC_Os02g42660 | OsDES1 |
|  | LOC_Os09g16920 | OsSLD1 |
| *Sorghum bicolor* | Sobic.002G045000 | SbFAD2.1 |
|  | Sobic.007G140800 | SbFAD6 |
|  | Sobic.001G407600 | SbFAD8.1 |
|  | Sobic.002G430100 | SbFAD8.2 |
|  | Sobic.008G002800 | SbFAD8.3 |
|  | Sobic.003G187800 | SbSLD1 |
|  | Sobic.004G260800 | SbFAD2.2 |
|  | Sobic.007G065600 | SbFAD4.1 |
|  | Sobic.008G005600 | SbFAD4.2 |

| **Species** | **Gene locus** | **Gene symbol** |
| --- | --- | --- |
| *Arabidopsis thaliana* | AT1G43800 | AtFAB2.1 |
|  | AT2G43710 | AtFAB2.2 |
|  | AT3G02630 | AtFAB2.3 |
|  | AT5G16240 | AtFAB2.4 |
|  | AT5G16230 | AtFAB2.5 |
|  | AT3G02610 | AtFAB2.6 |
|  | AT3G02620 | AtFAB2.7 |
| *Sorghum bicolor* | Sobic.006G048700 | SbFAB2.1 |
|  | Sobic.001G280700 | SbFAB2.2 |
|  | Sobic.001G265300 | SbFAB2.3 |
|  | Sobic.003G404000 | SbFAB2.4 |
|  | Sobic.002G258500 | SbFAB2.5 |
|  | Sobic.010G235300 | SbFAB2.6 |
|  | Sobic.001G334501 | SbFAB2.7 |
|  | Sobic.004G157550 | SbFAB2.8 |
|  | Sobic.003G377100 | SbFAB2.9 |
|  | Sobic.007G071600 | SbFAB2.10 |
|  | Sobic.004G153300 | SbFAB2.11 |
| *Glycine max* | Glyma.02G138100 | GmFAB2.1 |
|  | Glyma.07G207200 | GmFAB2.2 |
|  | Glyma.13G038600 | GmFAB2.3 |
|  | Glyma.13G038700 | GmFAB2.4 |
| *Oryza sativa* | LOC_Os01g65830 | OsFAB2.1 |
|  | LOC_Os01g69080 | OsFAB2.2 |
|  | LOC_Os02g30200 | OsFAB2.3 |
|  | LOC_Os03g30950 | OsFAB2.4 |
|  | LOC_Os03g53010 | OsFAB2.5 |
|  | LOC_Os04g31070 | OsFAB2.5 |
|  | LOC_Os08g09950 | OsFAB2.6 |
|  | LOC_Os08g10010 | OsFAB2.7 |
